# Neuropsychiatric symptoms: Risk factor or disease marker? A study of structural imaging biomarkers of Alzheimer's disease and incident cognitive decline

**DOI:** 10.1002/hbm.70016

**Published:** 2024-09-10

**Authors:** Dylan X. Guan, Tanaeem Rehman, Santhosh Nathan, Romella Durrani, Olivier Potvin, Simon Duchesne, G. Bruce Pike, Eric E. Smith, Zahinoor Ismail

**Affiliations:** ^1^ Graduate Science Education University of Calgary Calgary Alberta Canada; ^2^ Hotchkiss Brain Institute University of Calgary Calgary Alberta Canada; ^3^ Cumming School of Medicine University of Calgary Calgary Alberta Canada; ^4^ Quebec Heart and Lung Institute Québec City Quebec Canada; ^5^ Department of Radiology Université Laval Québec City Quebec Canada; ^6^ Department of Clinical Neurosciences University of Calgary Calgary Alberta Canada; ^7^ Department of Radiology University of Calgary Calgary Alberta Canada; ^8^ Department of Community Health Sciences University of Calgary Calgary Alberta Canada; ^9^ Department of Psychiatry University of Calgary Calgary Alberta Canada; ^10^ O'Brien Institute for Public Health University of Calgary Calgary Alberta Canada; ^11^ Clinical and Biomedical Sciences, Faculty of Health and Life Sciences University of Exeter Exeter UK

**Keywords:** Alzheimer's disease, dementia, magnetic resonance imaging, mild behavioral impairment, mild cognitive impairment, neuropsychiatric symptoms

## Abstract

Neuropsychiatric symptoms (NPS) are risk factors for Alzheimer's disease (AD) but can also manifest secondary to AD pathology. Mild behavioral impairment (MBI) refers to later‐life emergent and persistent NPS that may mark early‐stage AD. To distinguish MBI from NPS that are transient or which represent psychiatric conditions (non‐MBI NPS), we investigated the effect of applying MBI criteria on NPS associations with AD structural imaging biomarkers and incident cognitive decline. Data for participants (*n* = 1273) with normal cognition (NC) or mild cognitive impairment (MCI) in the National Alzheimer's Coordinating Center Uniform Data Set were analyzed. NPS status (MBI, non‐MBI NPS) was derived from the Neuropsychiatric Inventory Questionnaire and psychiatric history. Normalized measures of bilateral hippocampal (HPC) and entorhinal cortex (EC) volume, and AD meta‐region of interest (ROI) mean cortical thickness were acquired from T1‐weighted magnetic resonance imaging scans. Multivariable linear and Cox regressions examined NPS associations with imaging biomarkers and incident cognitive decline, respectively. MBI was associated with lower volume and cortical thickness in all ROIs in both NC and MCI, except for EC volume in NC. Non‐MBI NPS were only associated with lower HPC volume in NC. Although both of the NPS groups showed higher hazards for MCI/dementia than No NPS, MBI participants showed more rapid decline. Although both types of NPS were linked to HPC atrophy, only NPS that emerged and persisted in later‐life, consistent with MBI criteria, were related to AD neurodegenerative patterns beyond the HPC. Moreover, MBI predicted faster progression to dementia than non‐MBI NPS.

## Graphical abstract

Changes in behavior and personality (neuropsychiatric symptoms, NPS) that emerge and persist later in life are known as symptoms of mild behavioral impairment (MBI). We show that MBI is more strongly associated with neurodegenerative patterns linked to Alzheimer's disease than traditional ways of assessing NPS in older persons.

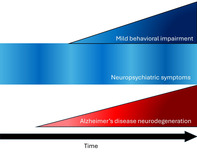

## 1 INTRODUCTION

Identifying older persons at early stages of neurodegenerative diseases, like Alzheimer's disease (AD), is crucial to mitigate the growing worldwide impact of dementia, for which AD is the most common cause (Prince et al., 2015). Early detection of preclinical and prodromal disease stages will facilitate planning for patient care and caregiving needs. Moreover, secondary prevention programs and disease‐modifying therapies may be most effective when administered early (Gauthier et al., 2016; Kivipelto et al., 2018).

Mild cognitive impairment (MCI) is widely recognized as an at‐risk state for dementia (Petersen, 2004). MCI diagnosis relies on self‐ or informant‐reported cognitive decline that is corroborated by objective cognitive testing, but with maintenance of functional independence (Albert et al., 2011). MCI is not synonymous with prodromal AD, as other neurodegenerative and non‐neurodegenerative conditions can present as MCI. However, it remains important to identify MCI for AD risk assessment, especially when biomarker confirmation of AD pathology is unavailable (Petersen et al., 2021). Since its formal conceptualization over two decades ago, the MCI construct has greatly facilitated AD research, underscoring the importance of early risk detection (Petersen, 2016). Clinical trials of AD disease‐modifying therapies now aim to slow the progression from MCI to syndromic AD dementia, with implications for clinical care.

While cognitive decline is the clinical hallmark of dementia, it frequently coexists with changes in behavior (Lyketsos et al., 2002). These neuropsychiatric symptoms (NPS) contribute to lower quality of life for patients and caregivers (Fischer et al., 2012; González‐Salvador et al., 2000). Analogous with cognition, milder NPS can also be observed before dementia onset and may signal early‐stage neurodegenerative disease. Mild behavioral impairment (MBI) was formally defined to capture these prodromal NPS (Ismail et al., 2016). MBI criteria identify NPS that are later‐life emergent and persistent, representing a change from longstanding behavior or personality, and which are not better explained by established psychiatric conditions. An a priori goal in development of MBI criteria was to leverage symptom natural history to help distinguish NPS prodromal to dementia from symptoms not associated with neurodegenerative etiologies, despite superficially similar clinical presentations (Ismail et al., 2016).

MBI, like MCI, can help facilitate scientific understanding of preclinical and prodromal stages of neurodegenerative diseases that lead to dementia (Creese & Ismail, 2022; Soto et al., 2024). When applied to MCI, MBI can improve specificity to further enrich samples for those likely to progress to dementia (Ismail, Leon, et al., 2023; Mortby, Black, et al., 2018). MBI has been linked to incident cognitive decline and dementia (Ismail et al., 2021; Kan et al., 2022; Rouse et al., 2024; Ruthirakuhan et al., 2022), and MCI participants with MBI are also significantly less likely to revert to normal cognition (NC) (McGirr et al., 2022). Additionally, a growing body of evidence has suggested an increasingly clearer link between MBI and AD biofluid and positron emission tomography (PET) biomarkers: greater amyloid‐β, phosphorylated tau, and neurofilament light pathological burden (Ghahremani et al., 2023; Ismail, Leon, et al., 2023; Johansson et al., 2021; Lussier et al., 2020; Miao et al., 2022; Naude et al., 2020; Naude et al., 2024).

Stringent application of MBI criteria yields greater specificity in differentiating neurodegenerative and non‐neurodegenerative NPS (Ghahremani et al., 2023; Ismail, Leon, et al., 2023; Naude et al., 2024; Showraki et al., 2019). In the same study where MBI predicted a lower reversion rate from MCI to NC, transient NPS did not (McGirr et al., 2022). Similar patterns were observed in AD biomarkers; MBI was linked to lower amyloid‐β 42/40 ratios and higher levels of phosphorylated and total tau, whereas transient NPS were only weakly associated with lower amyloid‐β 42/40 (Ismail, Leon, et al., 2023). Furthermore, the hazard of incident dementia among older adults with MBI was higher when symptoms were confirmed to be persistent as opposed to transient (Guan et al., 2023). Thus, just as MCI criteria help identify cognitive changes prodromal to dementia, MBI criteria help identify behavioral changes prodromal to dementia, particularly those prodromal to AD. Adding behavioral status to cognitive status improves dementia prognostication compared to cognitive status alone. These findings support the notion that both cognition and behavior are core dementia features that should be reported together, and, in the case of AD, both putatively driven by proteinopathies.

Although MBI and non‐MBI NPS (i.e., NPS that are transient or attributable to psychiatric conditions) have been compared in their associations with incident cognitive decline and AD biofluid biomarkers, their differences in terms of AD imaging biomarkers have not yet been thoroughly investigated. Moreover, previous MBI and non‐MBI NPS distinctions have predominantly focused on the symptom persistence criterion. Here, compared to non‐MBI NPS, we compared associations between MBI (defined by both symptom persistence and emergence de novo, i.e., not attributable to past psychiatric conditions) and AD structural neuroimaging AD biomarkers. Furthermore, in those with either NC or MCI at baseline, we compared NPS groups for hazard of incident cognitive decline and dementia.

## 2 METHODS AND MATERIALS

### 2.1 Study design

Data were acquired from the National Alzheimer's Coordinating Center (NACC), which consolidates clinical, neuroimaging, cognitive, behavioral, and functional data from over 30 National Institute on Aging‐funded Alzheimer's Disease Research Centers (ADRCs) across the United States (Beekly et al., 2004). The current analysis utilized data from participant visits occurring between June 2005 and February 2022 across 45 ADRCs. Where possible, participants were followed up approximately annually. All contributing ADRCs obtained ethics approval from their respective institutions prior to submitting data to NACC. Detailed descriptions of NACC recruitment and data collection procedures have been documented elsewhere (Beekly et al., 2004; Besser et al., 2018; Morris et al., 2006; Weintraub et al., 2009).

### 2.2 Participants

**FIGURE 1:**
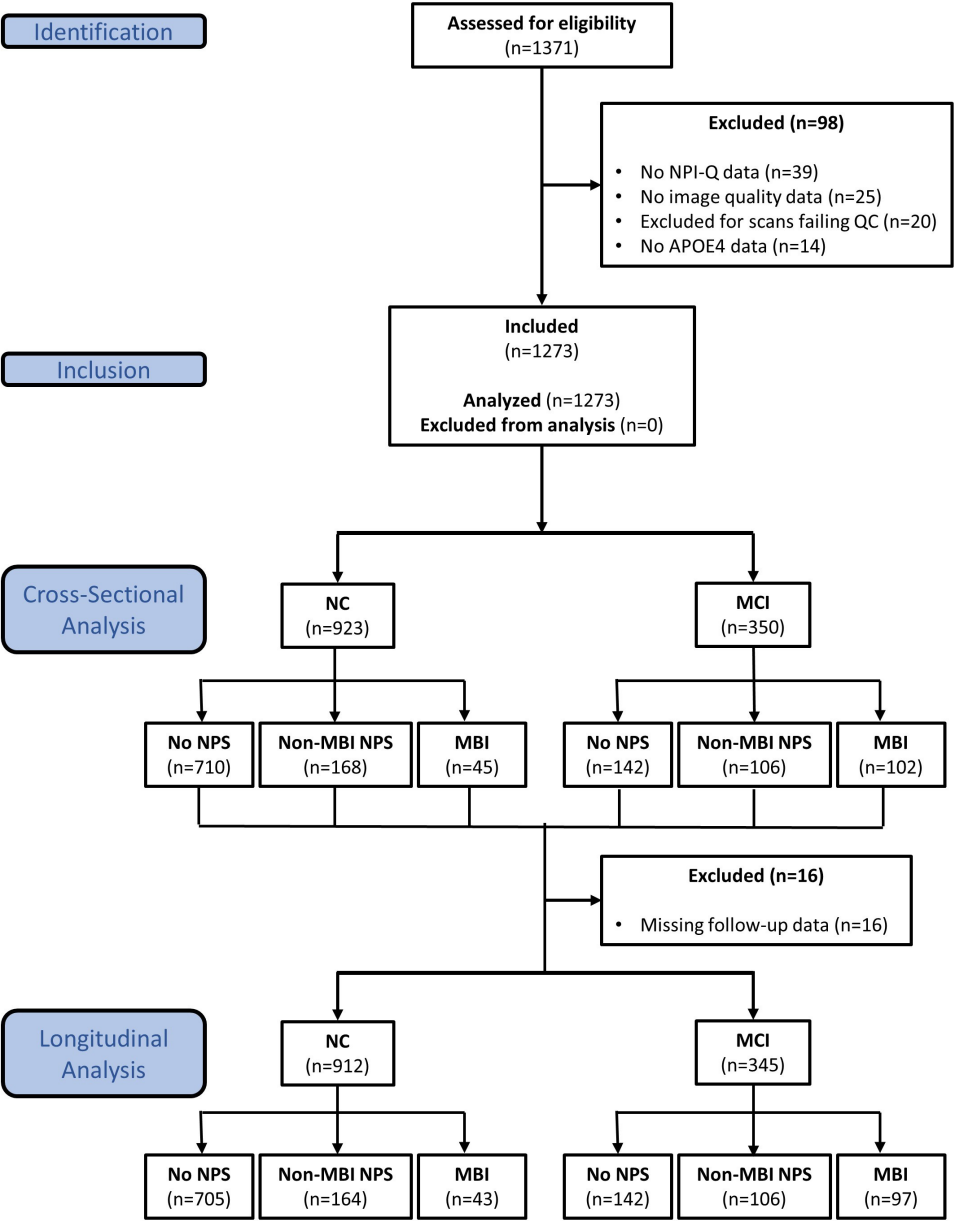
Participant flow diagram. MBI, mild behavioral impairment; MCI, mild cognitive impairment; NC, normal cognition; NPI‐Q, Neuropsychiatric Inventory Questionnaire; NPS, neuropsychiatric symptoms.

Baseline T1‐weighted scans of dementia‐free participants (*n* = 1371) were acquired from NACC servers. Adhering to the MBI diagnostic framework established by the Alzheimer's Association International Society to Advance Alzheimer's Research and Treatment (Ismail et al., 2016), we confirmed that no participants were aged <50 years. Although participants with a history of psychiatric conditions (e.g., posttraumatic stress disorder, bipolar disorder, schizophrenia, obsessive‐compulsive disorder, remote anxiety, or depression) are typically excluded from MBI case ascertainment, we included them in our study to have a broad representation of older persons with NPS, including those who met MBI criteria and those who did not. This approach simulates the clinical setting in which NPS are measured irrespective of psychiatric history. Additionally, we excluded participants missing Neuropsychiatric Inventory Questionnaire (NPI‐Q) data (*n* = 39), those without image quality measures required for normalization of structural neuroimaging measures (*n* = 25), those whose preprocessed scans failed quality control (*n* = 20), and without data for apolipoprotein E e4 (APOE4) alleles (*n* = 14). The final sample for cross‐sectional analysis consisted of 1273 participants, including 923 with NC and 350 with MCI (Figure 1). Of these participants, 1257 had longitudinal follow‐up data for survival analysis.

### 2.3 Measures

NPS were evaluated using the informant‐rated NPI‐Q (Cummings, 2020; Kaufer et al., 2000), which comprises 12 items, each representing a distinct domain (e.g., apathy, dsyphoria, agitation). NPS severity over the last month is rated from 0 to 3, with higher scores indicating greater severity. Because the NPI‐Q was initially designed to capture NPS in populations with dementia, it does not inherently distinguish between NPS that meet or do not meet MBI criteria. Therefore, we applied two validated algorithms to extrapolate MBI symptom severity and status (Guan et al., 2023; Mortby, Ismail, & Anstey, 2018; Sheikh et al., 2018).

Initially, we derived MBI domain scores from corresponding NPI‐Q subscales: decreased motivation (range = 0–3) from apathy; affective dysregulation (range = 0–9) from depression, anxiety, and elation; impulse dyscontrol (range = 0–9) from agitation, irritability, and aberrant motor behavior; social inappropriateness (range = 0–3) from disinhibition; and abnormal perception or thought content (range = 0–6) from delusions and hallucinations (Mortby, Ismail, & Anstey, 2018; Sheikh et al., 2018). The total MBI symptom severity (range = 0–30) was computed by summing these five domain scores, thereby reflecting global MBI symptom burden. As per MBI criteria, neurovegetative NPI‐Q items pertaining to nighttime behaviors and appetite/eating were excluded from the MBI score calculation (Ismail et al., 2016).

Next, we categorized participants at each visit according to three criteria: global MBI symptom severity score, history of psychiatric conditions (to operationalize the MBI de novo symptom emergence in later‐life criterion), and NPS presence in over two‐thirds of dementia‐free visits (to operationalize the MBI symptom persistence criterion). Previous data in NACC show that applying these criteria enhances MBI case ascertainment (Guan et al., 2023). Specifically, participants with an MBI score of 0 were considered to have No NPS. Participants with a score ≥1 but with a history of psychiatric conditions and/or symptom impersistence were classified as having non‐MBI NPS. Participants with persistent symptoms (score ≥1) not attributed to an existing psychiatric condition were classified as having MBI. More information about the operationalization of MBI in longitudinal datasets such as in NACC has been described previously (Guan et al., 2023).

### 2.4 MR image data acquisition and processing

T1‐weighted scans were preprocessed and segmented using the FreeSurfer version 6.0 processing pipeline (Fischl, 2012; Fischl et al., 2002). The pipeline performed motion correction, image registration into Talairach space, skull stripping, subcortical segmentation, intensity normalization, and grey‐white matter boundary tessellation. This procedure yielded estimates of grey matter volume and cortical thickness for 34 bilateral cortical regions as defined by the Desikan–Killiany cortical atlas (Desikan et al., 2006), and only volume for 18 bilateral subcortical structures (Fischl et al., 2002).

Given the large volume of scans, we executed all FreeSurfer pipelines on the Canadian Brain Imaging Research Platform (CBRAIN), a collaborative, web‐based research platform providing access to several high‐performance computing centers (Sherif et al., 2014). After processing, all images were visually inspected to identify structural abnormalities, imaging artifacts, and segmentation errors. Scans that showed repairable errors were manually edited and then reprocessed, whereas those with insufficient quality or irreparable errors were excluded from the analysis (*n* = 20; Figure 1). Excluded participants did not differ from other participants by age, sex, education, race, APOE4, or MBI status.

To account for variability in acquisition protocols across ADRCs and heterogeneity in scanner characteristics, which are known to impact structural neuroimaging measures (Kruggel et al., 2010), we employed the Normative Morphometry Image Statistics (NOMIS) tool (freely available online at https://github.com/medicslab/NOMIS) (Potvin et al., 2021). This tool normalizes measurements of grey matter volume and cortical thickness according to participant characteristics (age, sex, and intracranial volume) and image quality (voxel size/resolution, contrast‐to‐noise ratio, and surface reconstruction holes) based on a normative sample of nearly 7000 adults with NC. Thus, all measures of grey matter volume and cortical thickness are expressed as *Z* scores.

Structural neuroimaging measures of interest in the present study were volumes of the hippocampus (HPC) and entorhinal cortex (EC), along with mean cortical thickness of an AD meta‐ROI. This previously described meta‐ROI consists of the EC, inferior temporal gyrus, middle temporal gyrus, and fusiform gyrus (Jack Jr. et al., 2017). In this analysis, we considered only bilateral structural measures. These bilateral measures were determined by averaging normative grey matter volume or cortical thickness estimates across both hemispheres.

### 2.5 Statistical analysis

Sample characteristics were summarized using means, standard deviations (SDs), and ranges for continuous variables, and counts and percentages for categorical variables, stratified by cognitive status (NC vs. MCI). Differences between NC and MCI groups were assessed using independent samples *t*‐tests for continuous variables or chi‐square tests for categorical variables, as appropriate.

Multivariable linear regressions were used to model associations between the three‐level NPS grouping (exposure variable: No NPS [reference], non‐MBI NPS, MBI) and the structural neuroimaging measures (outcome variables). These structural measures included the bilateral HPC and EC grey matter volumes, as well as the mean cortical thickness of the AD signature meta‐ROI. Models adjusted for years of education, participant race/ethnicity, time elapsed between clinical and scanning visits, and the number of APOE4 alleles.

To validate MBI case ascertainment, we analyzed dementia‐free survival and incident cognitive decline (i.e., transition to MCI or dementia in NC participants; progression to dementia in MCI participants) across NPS group. Kaplan–Meier survival curves were first used to non‐parametrically estimate survival probability and median survival time, followed by Cox proportional hazards regressions to estimate adjusted hazard ratios (aHRs). Schoenfeld and Martingale residuals were inspected to verify that the proportional hazards and linearity assumptions were satisfied. The Cox regression models incorporated the same covariates, in addition to age, sex, baseline HPC and EC volumes, and mean cortical thickness of the AD meta‐ROI. NPS group status was handled as a time‐dependent exposure variable to account for the possibility that participants initially categorized as No NPS could develop non‐MBI NPS or MBI during the follow‐up period.

Both multivariable and survival analyses were conducted separately within NC and MCI cohorts to explore how these associations may change across different stages of the dementia continuum. Statistical significance for hypothesis tests related to the primary objective was defined using a threshold of *p* < .05. All analyses were conducted using R version 4.0.2 (R Core Team, 2020). Data access was granted by NACC following submission of an approved research proposal. Individuals interested in accessing the dataset utilized in this study are encouraged to direct requests to NACC.

## 3 RESULTS

### 3.1 Participant characteristics

Baseline characteristics of the 1273 included participants stratified by cognitive status are described in Table 1. On average, participants were predominantly female (60.3%) and identified as White (84.4%), with a mean ± SD age of 69.8 ± 9.8 years, and 15.8 ± 5.7 years of education. Notably, 39.3% of participants possessed one or two APOE4 alleles. Compared to those with NC, MCI participants were typically older, more frequently female, and had a higher prevalence of APOE4 alleles. No significant differences between the cognitive groups were observed in race/ethnicity or years of education completed.

**TABLE 1:** Participant characteristics stratified by cognitive status.

Variable	Total	NC	MCI	*p*
*n*	1273	923	350	
Age (years)	69.8 (9.8), 50–100	68.3 (10.0), 50–100	73.6 (8.0), 50–93	<.001
Sex (female)	768 (60.3)	602 (65.2)	166 (47.4)	<.001
Education (years)	15.8 (5.7), 0–99	15.9 (5.0), 2–99	15.6 (7.2), 0–99	.31
Race				.56
White	1074 (84.4)	781 (84.6)	293 (83.7)	
Asian	20 (1.6)	11 (1.2)	9 (2.6)	
Black	161 (12.6)	117 (12.7)	44 (12.6)	
Hispanic	1 (0.1)	1 (0.1)	0 (0)	
Indigenous	16 (1.3)	12 (1.3)	4 (1.1)	
Other	1 (0.1)	1 (0.1)	0 (0)	
Number of APOE4 alleles				<.001
0	772 (60.6)	596 (64.6)	176 (50.3)	
1	427 (33.5)	294 (31.9)	133 (38)	
2	74 (5.8)	33 (3.6)	41 (11.7)	
Past/current psychiatric history	412 (32.4)	297 (32.2)	115 (32.9)	.87
Posttraumatic stress disorder	8 (0.6)	5 (0.5)	3 (0.9)	.81
Bipolar disorder	2 (0.2)	0 (0)	2 (0.6)	.13
Schizophrenia	2 (0.2)	1 (0.1)	1 (0.3)	1
Remote anxiety	7 (0.5)	6 (0.7)	1 (0.3)	.72
Remote depression	259 (20.3)	200 (21.7)	59 (16.9)	.07
Other	134 (10.5)	85 (9.2)	49 (14.0)	.02
NPS status				<.001
No NPS	852 (66.9)	710 (76.9)	142 (40.6)	
Non‐MBI NPS	274 (21.5)	168 (18.2)	106 (30.3)	
MBI	147 (11.5)	45 (4.9)	102 (29.1)	
Decreased motivation	49 (3.8)	12 (1.3)	37 (10.6)	<.001
Affective dysregulation	102 (8)	32 (3.5)	70 (20)	<.001
Impulse dyscontrol	100 (7.9)	34 (3.7)	66 (18.9)	<.001
Social inappropriateness	31 (2.4)	8 (0.9)	23 (6.6)	<.001
Psychosis	9 (0.7)	3 (0.3)	6 (1.7)	.02
MBI symptom severity				
Global	1 (2.1), 0–21	0.6 (1.7), 0–21	2.1 (2.8), 0–15	<.001
Decreased motivation	0.1 (0.4), 0–3	0 (0.2), 0–3	0.3 (0.6), 0–3	<.001
Affective dysregulation	0.4 (0.9), 0–9	0.3 (0.8), 0–9	0.8 (1.2), 0–6	<.001
Impulse dyscontrol	0.4 (1.0), 0–7	0.3 (0.8), 0–6	0.8 (1.3), 0–7	<.001
Social inappropriateness	0.1 (0.4), 0–3	0 (0.3), 0–3	0.2 (0.5), 0–3	<.001
Psychosis	0 (0.3), 0–3	0 (0.2), 0–3	0.1 (0.4), 0–3	<.001

*Note*: All table values have been rounded to one decimal place, except for *p*‐values which have been rounded to two or three decimal places, as appropriate. Numeric variables are shown in mean (standard deviation), range. Categorical variables are shown in *n* (%). Statistical difference across cognitive groups were determined using independent samples *t*‐tests or chi‐squared tests, as appropriate. Remote depression and anxiety refer to episodes occurring more than 2 years ago.

Abbreviations: APOE4, apolipoprotein E e4; MBI, mild behavioral impairment; MCI, mild cognitive impairment; NC, normal cognition; NPS, neuropsychiatric symptoms.

Among all participants (72.5% NC, 27.5% MCI) at baseline, 11.5% exhibited MBI, with an average symptom severity score of 1.0 ± 2.1 (Table 1). The most prevalent MBI domains were affective dysregulation (8.0%) and impulse dyscontrol (7.9%), followed by decreased motivation (3.8%), social inappropriateness (2.4%), and psychosis (0.7%). Non‐MBI NPS were present in 21.5% of participants, of whom 59.9% had psychiatric history and 40.1% had impersistent NPS. Compared to participants with NC, participants with MCI more frequently reported both non‐MBI NPS and MBI and exhibited lower volumes in the HPC and EC, as well as thinner AD meta‐ROI.

### 3.2 Structural correlates of MBI

**FIGURE 2:**
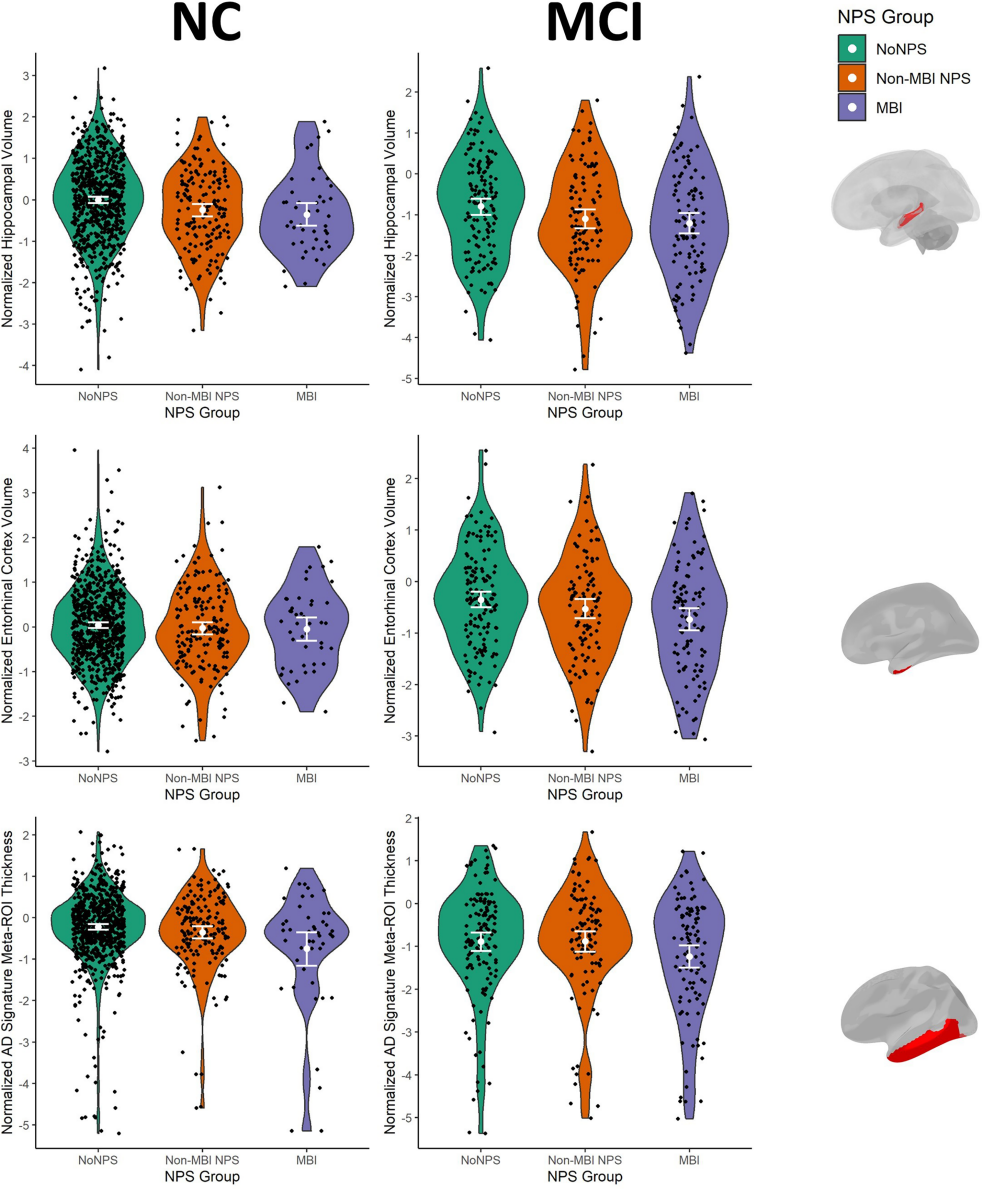
Violin plots of AD structural neuroimaging biomarkers as a function of NPS group. White dots indicate group means and error bars indicate 95% confidence intervals. Brain figures on the right show the anatomical location of the corresponding regions of interest. Although only one hemisphere is shown, bilateral measures were used for analysis. The AD signature meta‐ROI consisted of the entorhinal cortex, inferior temporal gyrus, middle temporal gyrus, and fusiform gyrus. AD, Alzheimer's disease; MBI, mild behavioral impairment; MCI, mild cognitive impairment; NC, normal cognition; NPS, neuropsychiatric symptoms; ROI, region of interest.

Among NC participants, those with non‐MBI NPS and those with MBI had HPC volumes that were on average 0.24 (95% CI: [−0.40, 0.08], *p* = .004) and 0.38 (95% CI: [−0.67, 0.09], *p* = .01) SD lower, respectively, compared to those with No NPS (Figure 2; Table 2). In NC, neither non‐MBI NPS (unstandardized *B* = −0.06, 95% CI: [−20.7, 0.09], *p* = .44) nor MBI (*B* = −0.08, 95% CI: [−0.35, 0.18], *p* = .54) were associated with EC volume. However, MBI was associated with a 0.50 SD (95% CI [−0.78, −0.21], *p* < .001) lower mean cortical thickness in the AD meta‐ROI. This pattern was not observed in those with non‐MBI NPS (*B* = −0.12, 95% CI: [−0.68, −0.24], *p* = .14).

**TABLE 2:** Mild behavioral impairment associations with AD structural neuroimaging biomarkers.

Outcome variable	NC			MCI		
Exposure variable	*β*	95% CI	*p*	*β*	95% CI	*p*
Hippocampus						
Non‐MBI NPS	−0.24	−0.40 to −0.08	**.004**	−0.28	−0.60 to 0.05	.09
MBI	−0.38	−0.67 to −0.09	**.01**	−0.40	−0.73 to −0.07	**.02**
Entorhinal cortex						
Non‐MBI NPS	−0.06	−0.21 to 0.09	.44	−0.17	−0.43 to 0.10	.22
MBI	−0.08	−0.35 to 0.18	.55	−0.37	−0.63 to −0.10	**.008**
AD meta‐ROI						
Non‐MBI NPS	−0.12	−0.28 to 0.04	.14	−0.04	−0.37 to 0.30	.83
MBI	−0.50	−0.78 to −0.21	**.001**	−0.39	−0.73 to −0.05	**.03**

*Note*: All table values have been rounded to two decimal places, with the exception of *p*‐values which have been rounded to two or three decimal places, as appropriate. All beta coefficients indicate normalized deviations in the corresponding structural measure (outcome variable) from a normative sample of NC older adults as a function of NPS group (exposure variable), after adjusting for years of education completed, participant race/ethnicity, time elapsed between clinical and scanning visits, and the number of APOE4 alleles. The AD signature meta‐ROI consisted of the entorhinal cortex, inferior temporal gyrus, middle temporal gyrus, and fusiform gyrus. Bolded *p*‐values indicate an association that met the statistical significance threshold of *p* < .05.

Abbreviations: 95% CI, 95% confidence interval; AD, Alzheimer's disease; APOE4, apolipoprotein E e4; MBI, mild behavioral impairment; MCI, mild cognitive impairment; NC, normal cognition; NPS, neuropsychiatric symptoms; ROI, region of interest.

In the MCI group, MBI was associated with all three structural neuroimaging measures of interest: HPC volume (*B* = −0.52, 95% CI: [−0.88, −0.17], *p* = .004), entorhinal volume (*B* = −0.40, 95% CI: [−0.68, −0.11], *p* = .006), and AD meta‐ROI mean cortical thickness (*B* = −0.38, 95% CI: [−0.73, −0.05], *p* = .03). In contrast, MCI participants with non‐MBI NPS showed no significant associations with any of the AD structural biomarkers: HPC volume (*B* = −0.28, 95% CI [−0.60, 0.05], *p* = .09), entorhinal volume (*B* = −0.17, 95% CI [−0.43, 0.10], *p* = .22), and AD meta‐ROI mean thickness (*B* = −0.04, 95% CI [−0.37, 0.30], *p* = .83).

### 3.3 MBI and incident cognitive decline

Across the cohort, 58.9% of MCI cases and 85.3% of dementia cases were given an AD etiological diagnosis, respectively. Kaplan–Meier survival analysis revealed that nearly half (46.5%) of NC participants with MBI experienced cognitive decline (i.e., MCI) within a median time of 9.97 years. In contrast, fewer non‐MBI NPS (21.3%) and No NPS (20.9%) participants developed MCI or dementia during the follow‐up period. A similar pattern was observed within the MCI group, who were already at higher risk for dementia. Among MCI participants, 70.1% of those with MBI progressed to dementia, in comparison to 63.2% of non‐MBI NPS participants and 46.5% of those with No NPS. The shortest median time to dementia was observed in the MBI group, at 2.20 years, followed by the non‐MBI NPS group (3.94 years), and the No NPS group (7.98 years; *p* < .001). Within MBI participants with NC or MCI at baseline who progressed to dementia, 87.2% were given an AD etiological diagnosis, 5.1% were given a Lewy body dementia (LBD) etiological diagnosis, and the remaining 7.7% were given a non‐AD and non‐LBD diagnosis.

**FIGURE 3:**
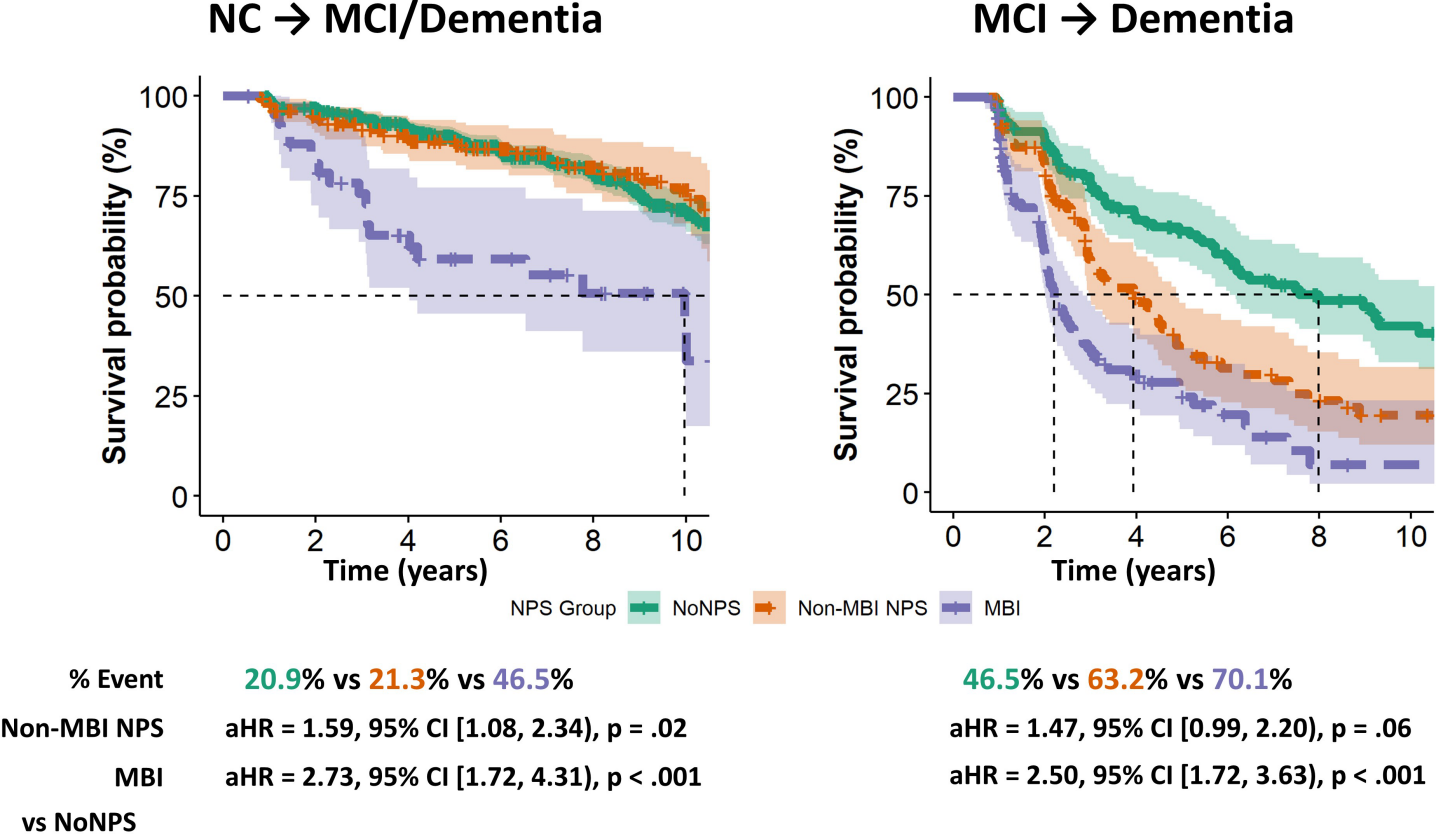
Survival analyses of incident mild cognitive impairment and/or dementia as a function of NPS group. Dashed lines in the Kaplan–Meier survival curves indicate median time to event. Hazard ratios shown below the Kaplan–Meier survival curves were derived from Cox proportional hazard regression models adjusting for age, sex, years of education completed, participant race/ethnicity, time elapsed between clinical and scanning visits, and the number of APOE4 alleles, baseline volumes of the bilateral hippocampal and entorhinal cortex, and the mean cortical thickness of the AD meta‐ROI. 95% CI, 95% confidence interval; AD, Alzheimer's disease; aHR, adjusted hazard ratio; APOE4, apolipoprotein E e4; MBI, mild behavioral impairment; MCI, mild cognitive impairment; NC, normal cognition; NPS, neuropsychiatric symptoms; ROI, region of interest.

The Cox regression models, adjusting for non‐imaging covariates (i.e., age, sex, education, race, APOE4), showed that NC participants with MBI had a 2.83‐fold higher hazard (95% CI: [1.80, 4.44], *p* < .001) for developing MCI or dementia compared to No NPS. This hazard was intermediate in those with non‐MBI NPS (aHR = 1.54, 95% CI: [1.05, 2.26], *p* = .02). Similar patterns were observed in the MCI cohort; participants with MBI and non‐MBI NPS have HRs for progression of 3.11 (95% CI: [2.15, 4.50], *p* < .001) and 1.63 (95% CI: [1.09, 2.43], *p* = .002), respectively. Even after further accounting for AD structural biomarkers, including HPC and entorhinal volumes and AD meta‐ROI mean thickness, the HRs for MBI and non‐MBI NPS remained largely consistent (Figure 3).

## 4 DISCUSSION

Among 1273 participants with NC or MCI, MBI was associated with lower volume or mean cortical thickness in all three examined AD structural biomarkers of interest, with the exception of EC volume in those with NC. In contrast, non‐MBI NPS was associated only with lower HPC volume in NC participants. We also observed that both MBI and non‐MBI NPS predicted accelerated progression to cognitive decline and dementia. However, the hazard of progression was higher in those with MBI compared to those with non‐MBI NPS.

This study is not the first to observe MBI associations with medial temporal lobe atrophy. Prior research has consistently shown lower entorhinal and HPC volumes in older adults with MBI (Matuskova et al., 2021; Shu et al., 2021), as well as with individual domains such as impulse dyscontrol (Gill et al., 2021). A recent systematic review further identified the HPC and EC among several neural correlates of MBI (Matsuoka et al., 2023). The present study contributes novel insights by directly comparing these MBI relationships to those observed in individuals with non‐MBI NPS. Additionally, our study replicates previous findings that MBI is more strongly associated with incident cognitive decline and dementia than non‐MBI NPS (Ghahremani et al., 2023; Ismail, Leon, et al., 2023; Ismail et al., 2021; McGirr et al., 2022).

Understanding the difference between dementia risk factors and disease markers is key to interpreting these data. While both terms denote higher likelihood of future dementia in this context, risk factors influence the incidence or progression of underlying diseases that lead to dementia, acting as external modifiers (Ganguli & Kukull, 2010). Psychiatric conditions are examples of risk factors, which can arise independently of neurodegenerative processes, and were present in nearly two‐thirds of non‐MBI NPS participants in this study. In contrast, disease markers are overt clinical symptoms or signs that the underlying disease process has already begun, that is, reverse causality. Applying the MBI criteria of later‐life emergent and persistent changes in behavior selects out the NPS group most likely to have underlying neurodegenerative disease, with the remaining NPS either representing risk factors for dementia or symptoms secondary to stressors and life events. Thus, MBI represents an improvement over conventional uses of NPS in dementia prognostication. These findings are consistent with recent tau‐PET imaging data for global MBI (Naude et al., 2024), and longitudinal data on apathy, affect, and psychosis, when comparing application of MBI criteria versus conventional approaches (Ebrahim et al., 2023; Ismail, Ghahremani, et al., 2023; Vellone et al., 2022).

The association between non‐MBI NPS and lower HPC volume in NC aligns with its classification as a dementia risk factor (Elser et al., 2023; Livingston et al., 2020). Indeed, this association may hint at one mechanism through which psychiatric conditions elevate the risk of dementia. Previous studies have observed reduced HPC volume in common psychiatric conditions, such as major depressive disorder (Campbell et al., 2004; Videbech & Ravnkilde, 2004). These individuals may consequently have lower brain reserve, particularly in regions critical for memory, like the HPC, leading to steeper cognitive decline once afflicted with AD. The association between non‐MBI NPS and HPC volume might weaken after participants transition to MCI, as AD may contribute to HPC atrophy independently of psychiatric conditions. Further aligning with classification as a risk factor, non‐MBI NPS were not correlated with structural alterations in other regions vulnerable to AD, including the EC and AD meta‐ROI. In contrast, MBI was associated with changes not only in the HPC across NC and MCI, but also in the EC and AD meta‐ROI. These data suggest that, while both types of NPS are linked to HPC atrophy, only NPS that emerge and persist later in life are related to AD neurodegenerative patterns extending beyond the HPC.

Although MBI was associated with lower EC volume in MCI, this association was not observed in NC. Braak staging posits that neurofibrillary tangle accumulation in AD begins in the transentorhinal cortex, followed by the EC and HPC (Braak & Braak, 1991; Braak & Braak, 1995). We raise several potential explanations for this discrepancy. First, Braak staging was based on the presence of tau aggregates rather than the extent of neuronal atrophy, which usually occurs downstream from tau buildup. Second, Braak staging was described through postmortem neuropathologic examinations of AD patients and may not precisely correspond to in vivo structural neuroimaging measurements. Third, multiple studies report that HPC volumes may be measured more reliably than entorhinal volumes (Juottonen et al., 1999; Xu et al., 2000), which may be especially important during the earliest AD stages, such as in NC, where atrophy is minimal. Finally, when evaluating structural biomarkers of AD that involve the EC, measuring mean cortical thickness across an AD meta‐ROI is generally preferred (Schwarz et al., 2016). Consistent with this, our study found an association between MBI and the meta‐ROI mean cortical thickness measure in both NC and MCI, while non‐MBI NPS did not.

We found lower volume and thickness in key AD regions among MBI participants. However, it is important to exercise caution against drawing causal inferences from these data, particularly the notion that MBI is a direct manifestation of early‐stage AD structural changes. Prevailing models of the AD pathological cascade posit that changes in neurodegenerative biomarkers and cognition are preceded by amyloid β and tau proteinopathies, often by years if not decades (Jack Jr. et al., 2010). A growing body of evidence has linked MBI to these earlier biomarkers. The Canadian TRIAD study found correlations between greater MBI symptom severity and elevated amyloid‐PET tracer uptake in NC participants (Lussier et al., 2020). The Swedish BioFINDER2 study demonstrated that NC older adults with MBI exhibited greater tau‐PET tracer uptake in the EC and HPC (Johansson et al., 2020). Similarly, a recent ADNI analysis of NC and MCI participants demonstrated tau‐PET tracer uptake in early Braak stage ROIs in MBI, but not for non‐MBI NPS (Naude et al., 2024). Additionally, recent studies have linked MBI to lower amyloid beta 42/40 (Ismail, Leon, et al., 2023; Miao et al., 2022) and higher levels of phosphorylated tau‐181 (Ghahremani et al., 2023; Ismail, Leon, et al., 2023). Emerging research also indicates a potential link between MBI and reduced integrity of the locus coeruleus (Cassidy et al., 2022), a brainstem region hypothesized to be affected by AD tau pathology even in advance of medial temporal lobe tauopathy (Braak et al., 2011). Finally, a functional MRI study found associations between MBI and changes in default mode network and salience network connectivity, consistent with early AD changes (Ghahremani, Nathan, et al., 2023). It is clear that more research is needed to better understand the relationship between MBI and various pathological changes that precede or accompany neurodegeneration. Future studies should also investigate how synaptic dysfunction, vascular pathology, and other neurodegenerative co‐pathologies may relate to MBI.

MBI case ascertainment remains a notable limitation of this study despite the use of two validated algorithms to derive MBI status from longitudinal NPI‐Q data (Guan et al., 2023; Mortby, Ismail, & Anstey, 2018; Sheikh et al., 2018). While the MBI criteria require symptom persistence for more than 6 months (Ismail et al., 2016), the NPI‐Q only has a reference range of 1 month (Cummings, 2020; Kaufer et al., 2000). The MBI Checklist, which has a 6‐month reference range, was developed to identify a broad array of mild NPS seen in advance of dementia (Hu et al., 2023; Ismail et al., 2017). MBI‐C use in future studies may help overcome the case ascertainment limitation. Potential misclassification of participants in the non‐MBI NPS group is another limitation: MBI may be misclassified as a psychiatric condition, especially if natural history is not well‐considered during diagnostic assessment. Indeed, misdiagnosis of MBI is common, with patients funnelled to geriatric psychiatry services until neurodegenerative disease declares itself (Matsuoka et al., 2019). Other studies of MBI may benefit from taking in account whether psychiatric conditions are of recent or remote onset to address this concern. Furthermore, measurement of NPS may differ between self‐ and informant ratings, the latter of which were used in this study (Creese et al., 2020). Whether NPS associations with AD biomarkers vary based on the source of NPS information should be explored further.

Notably, our analysis only examined three ROIs based on a hypothesis‐driven approach. However, other neuroimaging studies of MBI have identified structural changes in several brain regions outside the medial temporal lobe (Shu et al., 2021; Yoon et al., 2022). Utilizing voxel‐based morphometry may yield a more comprehensive understanding of the structural changes associated with MBI. We also did not examine structural neural correlates of individual MBI domains. Although studies have shown that MBI domains are differentially associated with cognitive decline (Agüera‐Ortiz et al., 2022; Creese et al., 2023; Ebrahim et al., 2023; Ismail, Ghahremani, et al., 2023; Vellone et al., 2022), further research is necessary to determine whether MBI domains have similar or distinct associations with structural AD biomarkers.

In conclusion, we provide evidence from structural neuroimaging that NPS meeting MBI criteria in persons with NC and MCI are more closely associated with AD patterns of neurodegeneration than are non‐MBI NPS. Further, MBI has a greater hazard of incident cognitive decline and dementia, primarily AD. These findings underscore the importance of incorporating natural history into NPS determination, specifically for later‐life emergence and persistence of symptoms, to distinguish between dementia risk factor and neurodegenerative disease marker. This distinction can facilitate dementia research and improve clinical trial sample enrichment.

## AUTHOR CONTRIBUTIONS

**Dylan X. Guan**: Conceptualization; software; formal analysis; writing—original draft; visualization. **Tanaeem Rehman**: Conceptualization; data curation; formal analysis; writing—review & editing. **Santhosh Nathan**: Data curation; writing—review & editing. **Romella Durrani**: Data curation; writing—review & editing. **Olivier Potvin**: Software; writing—review & editing. **Simon Duchesne**: Software; writing—review & editing. **G. Bruce Pike**: Writing—review & editing. **Eric E. Smith**: Writing—review & editing. **Zahinoor Ismail**: Conceptualization; resources; data curation; writing—review & editing; supervision.

## CONFLICT OF INTEREST STATEMENT

Eric E. Smith reports consulting (unpaid) for Alnylam Pharmaceuticals and Eli Lilly, and an advisory board (unpaid) for Eisai. Zahinoor Ismail has served as a consultant/advisor to Eisai, Lilly, Lundbeck/Otsuka, Novo Nordisk, and Roche. The remaining authors declare no conflicts of interest.

## Data Availability

The data that support the findings of this study are available from NACC. Individuals interested in accessing the dataset utilized in this study are encouraged to direct requests to NACC.
